# Does *Cimicifuga racemosa* have the effects like estrogen on the sublingual gland in ovariectomized rats?

**DOI:** 10.1186/s40659-017-0115-x

**Published:** 2017-03-14

**Authors:** Yun-Meng Da, Kai-Yu Niu, Shu-Ya Liu, Ke Wang, Wen-Juan Wang, Jing Jia, Li-Hua Qin, Wen-Pei Bai

**Affiliations:** 1 Department of Stomatology, General Hospital of Chinese People’s Armed Police Forces, NO.69 of Yongding Road, Haidian District, Beijing, China; 2 Department of Stomatology, Hebei Provincial Eye Hospital, NO. 399 of Quannan East Street, Qiaodong District, Xingtai, Hebei China; 3 Department of Anatomy, Peking University Health Science Center, School of Basic Medicine, Beijing, China; 4 Beijing Shijitan Hospital, Capital Medical University, 10 Tieyi Road, Haidian District, Beijing, China

**Keywords:** Estradiol, *Cimicifuga racemosa*, Sublingual gland, Subcellular structure, Caspase-3

## Abstract

**Background:**

*Cimicifuga racemosa* is one of the herbs used for the treatment of climacteric syndrome, and it has been cited as an alternative therapy to estrogen. Apart from hectic fevers, dyspareunia and so on, dry mouth also increase significantly after menopause. It has not yet been reported whether *C. racemosa* has any impact on the sublingual gland, which may relate to dry mouth. In an attempt to determine this, we have compared the effects of estrogen and *C. racemosa* on the sublingual gland of ovariectomized rats.

**Results:**

HE staining showed that the acinar cell area had contracted and that the intercellular spaces were broadened in the OVX (ovariectomized rats) group, while treatment with estradiol (E2) and iCR (isopropanolic extract of *C. racemosa*) improved these lesions. Transmission electron microscopy showed that rough endoplasmic reticulum expansion in mucous and serous acinar epithelial cells and apoptotic cells was more commonly seen in the OVX group than in the SHAM (sham-operated rats) group. Mitochondria and plasma membrane infolding lesions in the striated ducts were also observed. These lesions were alleviated by both treatments. It is of note that, in the OVX + iCR group, the volume of mitochondria in the striated duct was larger than in other groups. Immunohistochemical staining showed that the ratio of caspase-3 positive cells was significantly increased in the acinar cells of the OVX group compared with the SHAM group (p < 0.05); and the MA (mean absorbance) of caspase-3 in the striated ducts also increased (p < 0.05). Estradiol decreased the ratio of caspase-3 positive cells and the MA of caspase-3 in striated ducts significantly (p < 0.05). ICR also reduced the ratio of caspase-3 positive cells and the MA in the striated ducts (p < 0.05), but the reduction of the MA in striated ducts was inferior to that of the OVX + E2 group (p < 0.05).

**Conclusion:**

Both estradiol and iCR can inhibit subcellular structural damage, and down-regulate the expression of caspase-3 caused by ovariectomy, but their effects were not identical, suggesting that both drugs confer a protective effect on the sublingual gland of ovariectomized rats, but that the specific location and mechanism of action producing these effects were different.

**Electronic supplementary material:**

The online version of this article (doi:10.1186/s40659-017-0115-x) contains supplementary material, which is available to authorized users.

## Background

Menopause is a physiological process, which is delineated by cessation of menstruation for more than 6 months, resulting from a natural or surgical loss of ovarian function. It typically occurs in the fifth decade of life in women [1]. Menopausal syndrome includes hectic fevers, night sweats, dyspareunia, dry mouth and some other common symptoms. It’s worth noting that, the incidence rate of dry mouth increases significantly. Estrogen treatment can improve postmenopausal symptoms of dry mouth. One study found that in postmenopausal women (61–76 years old) estrogen treatment for 1 year significantly improved labial gland salivary flow rate, total salivary secretion rate and buffering capacity [2]. The WHI (Women’s Health Initiative) noted that long-term use of estrogen may increase the risk of heart disease, strokes, blood clots and breast cancer. Recently, clinical data has shown that in postmenopausal women unwilling to receive continuous HRT (hormone replacement therapy), 50% will choose alternative therapies to alleviate menopausal symptoms [3–6].

*Cimicifuga racemosa,* also named black cohosh, is an herb belonging to the buttercup family, which grows in North America. In Europe, it has been used for over 50 years for the treatment of climacteric syndrome [7, 8]. A variety of drugs currently available use black cohosh as the main ingredient, including Klimadynon^®^ and Remifemin. When the therapeutic effects of black cohosh and tibolone, a synthetic selective tissue estrogenic activity regulator (STEAR), has estrogenic, progestogenic, and androgenic effects, on climacteric syndrome were compared, the efficacy of black cohosh was found to be equal to that of tibolone, and the incidence of adverse reactions was lower [9]. Further, black cohosh can be used as an alternative medicine to estrogen for treating menopausal osteoporosis, without causing adverse effects on the breast and uterus [10]. A methanol extract of black cohosh was found to be an antioxidant capable of protecting cells from DNA damage caused by reactive oxygen species, and cleaned free-radicals of 1,1-diphenyl-2-picryl-hydrazyl (DPPH). Also, complexes with antioxidant activity were isolated, including caffeic acid, ferulic acid, cimiracemate A, cimiracemate B, petasites acid etc., which can inhibit menadione-mediated DNA damage in S30 breast cancer cells in vitro [11]. It has been reported that isoimperatorin, *Cimicifuga* glycoside E and 23-*O*-acetylshengmanol-3-xyloside extracts from black cohosh inhibited tumour necrosis factor alpha (TNF-α)-mediated vascular cell adhesion protein-1 (VCAM-1) expression in human epithelial cells by suppressing the phosphorylation of peroxisome proliferator-activated receptor gamma (PPAR-γ), extracellular regulated kinase 1 and 2 (ERK1/2) and protein kinase C (PKC) signaling molecules [12].

This was the first study to investigate the effects of *C. racemosa* on sublingual gland, the only salivary gland playing the main role in the secretion of mucus which is an important ingredient in keeping oral cavity lubrication. It is well known that both the cell morphology change and apoptosis were the key events in postmenopausal women. Since the discovery that activated caspase-3 changes apoptotic cell structure and causes DNA splitting and cell atrophy, it has been broadly recognized that caspase-3 is a histochemical marker specific for cell apoptosis. Therefore this study is aims to analyze the effects of black cohosh in comparison to estradiol in morphology and caspase-3 protein levels of ovariectomized rats. Taking into account the complex components of black cohosh extract, investigating its effects on the sublingual gland will help to elucidate the mechanisms of action, and its clinical applications.

## Methods

### Animals

Twenty adult female rats (Sprague–Dawley), purchased from the Department of Laboratory Animal Science of Peking University Health Science Center, which were 8–10 weeks old and weighed 240–280 g, were used for the study. The rats were maintained under controlled conditions (25 ± 1 °C, room temperature, with a light/dark cycle of 12 h and 40–50% humidity), and fed with soy-free forage and given free access to water under indirect light for 2 weeks. The study was approved by the local Ethics Committee, and all experiments were conducted in conformity with the National Institutes of Health Guidance for the Care and Use of Laboratory Animals. The study was also approved by the Biomedical Ethics Committee of Peking University (approval number: LA2012–82).

### Reagents and instruments

Rabbit anti-rat caspase-3 polyclonal antibodies (#ZS-7148) and a streptavidin/peroxidase staining kit (Beijing Zhongshan Golden Bridge Biotechnology Co. Ltd. Beijing, China) were used for immunohistochemistry. Commercially available estradiol valerate (Bujiale; 1 mg per tablet, Bayer Health Care Co. Ltd, Batch number 256A 2; Guangzhou, China) and *C. racemosa* (Remifemin tablets; 20 mg per tablet, Schaper & Brümmer Ltd & Co KG, Batch number 123813; Germany) extracted using 40% isopropyl alcohol were used in this study.

### Establishment of the ovariectomized rat model

Experimental groups were established as follows: a sham-operated group (SHAM, n = 5), an ovariectomized group (OVX, n = 5), an OVX group treated with estradiol (E2) valerate (OVX + E2, n = 5), and an OVX group treated with the iCR (OVX + iCR, n = 5). Firstly, ovariectomy was performed on rats under general anesthesia. Following appropriate asepsis and antisepsis, the abdominal cavity was exposed using a scalpel. Instead of the ovaries, an equal quantity of fat from around the ovaries was resected from the SHAM group. In the other groups, bilateral ovaries were totally removed. Finally, the abdominal cavity was closed with a 3-0 seam interrupted suture. The exfoliated vaginal cells, changing in cell morphology and types with fluctuations in estrogen levels, were observed for 7 consecutive days from the third day after surgery to verify successful OVX [13]. Two weeks were allowed after the operation for wound healing, and then rats were given daily gavage for 4 weeks [14, 15].

### Dosage and administration

The preparation of experimental medicines was as follows: the estradiol valerate and *C. racemosa* tablets were dissolved in distilled water by sonication to form a uniform suspension. The concentration of estradiol was 0.2 g/l and of iCR, 12 g/l. Gavage was administered to all rats from 8:30 to 9:30 a.m. Doses were as follows: the SHAM group received distilled water (10 ml/kg); the OVX group received distilled water (10 ml/kg); the OVX + E2 group received estradiol valerate (0.8 mg/kg) and the OVX + iCR group received *C. racemosa* (60 mg/kg) [13, 16]. Rats were weighted on alternate days and the dose was adjusted according to any change in body weight.

### Sample collection

To avoid the normal fluctuation of estrogen affects the results, the sample collection of SHAM group was performed in estrus used the vaginal smear method. Following sodium pentobarbital (40 mg/kg intraperitoneal injection) anesthesia, tissues were first collected for electron microscopy. The neck skin and fascia were cut with scissors, revealing the sublingual glands. A small part of the left gland was cut away with a sharp blade and cut into pieces of approximately 1 mm^3^. These were fixed in 3% v/v glutaraldehyde for 2 h. Tissues were washed with 0.1 mol/l phosphate-buffered (PB) buffer, fixed in osmium tetroxide for 2 h and then, following dehydration in ethanol solutions of increasing concentration, embedded in Epok 812. Ultrathin sections (70 nm thick) were taken and stained with uranyl acetate and lead citrate. These were then examined by electron microscopy (JEM-2100). Each image was viewed at the same brightness and contrast for production of photomicrographs, in order to compare changes in the rough endoplasmic reticulum, mitochondria and secretory granules.

After collection of tissues for electron microscopy, the heart was exposed, a perfusion catheter was inserted into the ascending aorta and the right atrium was incised. Perfusion was carried out using 200 ml normal saline (0.9% g/ml sodium chloride aqueous solution) and 300 ml 4% g/ml paraformaldehyde solution (paraformaldehyde dissolved in 0.1 mol/l PB). After perfusion, the right sublingual gland was immediately removed and post-fixed in 4% g/ml paraformaldehyde solution for 24 h. After dehydrated, the tissue was embedded in paraffin. Three serial sections of a sample, 5.0 μm in thickness, were mounted on each microscope slide, and 20 slides were collected of per animal. After drying, the paraffin sections were stained with hematoxylin and eosin (HE).

### Immunohistochemical staining

Paraffin-embedded sections were heated to 60 °C to melt the paraffin, then washed in xylene and rehydrated by graded washing in 100, 95, 80, 70, 50% ethanol in distilled water (each concentration for 20 min). Sections were then incubated in 0.3% Triton X-100 at 37 °C for 30 min, and, for antigen retrieval, placed in a boiling water bath for 15 min. They were next placed in 0.3% hydrogen peroxide for 15 min. The sections were washed with 0.01 mol/l phosphate-buffered saline (PBS; pH 7.4, 4 °C) (3 × 5 min) between each step. Sections were then incubated in biotinylated goat anti-rabbit IgG serum (Beijing Zhongshan Goldenbridge Biotechnology Co., Ltd. Beijing, China) for 2 h, and then with rabbit anti-rat caspase-3 polyclonal antibody (1:100 dilution in 0.01 mol/l PBS; Beijing Zhongshan Golden Bridge Biotechnology Co, Ltd. Beijing, China) at room temperature for 1 h and then at 4 °C overnight. The following day, the sections were placed at room temperature for 1 h, and then avidin–biotin complex (ABC) staining kit (Beijing Zhongshan Golden Bridge Biotechnology Co. Ltd. Beijing, China) was used. Finally, each section was stained with DAB, using a staining kit (Beijing Zhongshan Golden Bridge Biotechnology Co. Ltd. Beijing, China), for approximately 5 min, and the immunoreactive products were visualized as a brown stain. PBS (0.01 mol/l) was used for washing between each step (3 × 5 min). Then, hematoxylin was used to stain the cell nuclear in order to facilitate observation. After dehydration and mounting of neutral resins, sections were observed under a light microscope (Leica; BX51). The same process was used for negative controls, except that 0.01 mol/l PBS was used instead of rabbit anti-rat caspase-3 polyclonal antibodies.

### Data collection and statistics

#### The percentage of the gap area

Under the same conditions of brightness and contrast (at 200× magnification), five fields of vision were randomly selected from each HE film. Image-Pro Plus 6.0 software was used. Firstly, the non-staining acini was filled with red color manually in order to distinct with the gap, and then the area of the gap and the total area of the lobule were calculated used Count/Size instrument of Image-Pro Plus 6.0 software. The percentage of the gap area was then obtained by dividing the area of the gap by the total area (Additional file 1).

#### Percentage of caspase-3 positive cells

At a magnification of 400×, five fields of vision were randomly selected from each immunohistochemical film: “a”, represents the number of positive acinar cells in each photo; “b” is the total number of acinar cells in the image; “a/b” is the percentage of positive cells in each film. A mean of the data for the five fields of vision was taken to obtain the ratio of positively stained cells to the total cells in each rat (Additional file 2).

#### Average optical density (mean absorbance, MA) of caspase-3 immunoreactivity in the striated ducts

Under the same conditions of brightness and contrast (at 400× magnification), five fields of vision were randomly selected from each immunohistochemical film. Image-Pro Plus 6.0 software was used to analyze the MA. Firstly, the area of striated ducts was measured manually (area of interest, AOI), and then the total area and the integral absorbance (IA) of AOI were calculated used Count/Size instrument of Image-Pro Plus 6.0 software; MA was then obtained by dividing IA by the total area (Additional file 3).

All the data were analyzed using SPSS17.0 and recorded as means ± standard deviations (x ± SD). To analyse caspase-3 expression, a one-way analysis of variance (one-way ANOVA) was performed, followed by an LSD post hoc test.

## Results

### HE staining

**Fig. 1:**
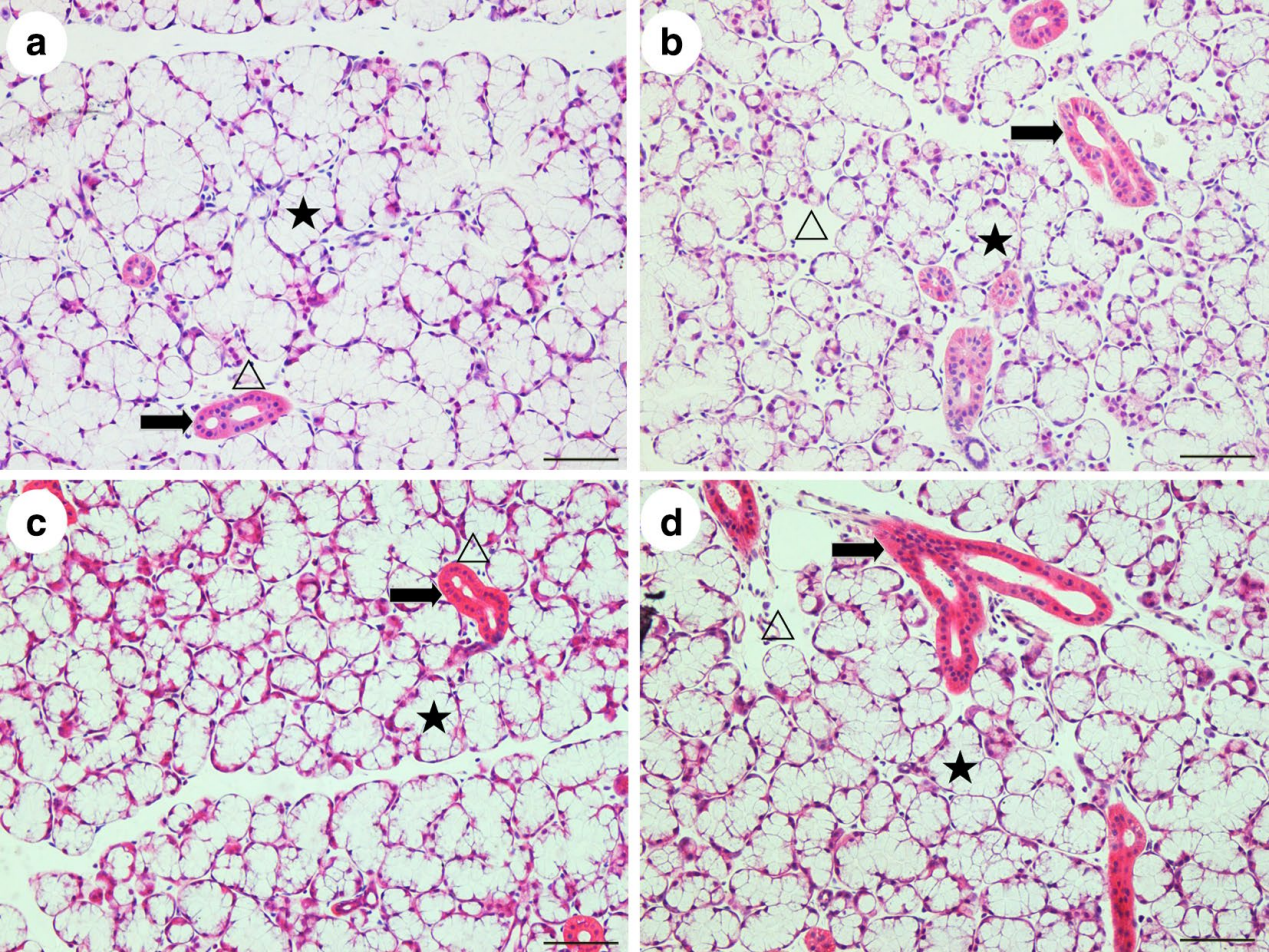
HE staining of sublingual glands. **a** SHAM group; **b** OVX group; **c** OVX + E2 group; **d** OVX + iCR group. The acini were shriveled and had broader gaps between them in **b** compared with **a**, **c** and **d**. *Triangle* denotes the broadened gap. *Star* denotes the acinus. The *black arrows* denote the striated duct. *Bars* 200 μm

**Fig. 2:**
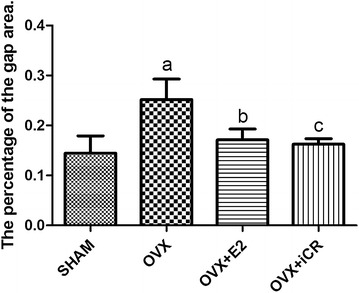
The percentage of the cell gap area of four groups. Values are given as mean ± SD. ^a^p < 0.05 for SHAM vs. OVX. ^b,c^p < 0.05 for OVX vs. OVX + E2 and OVX + iCR respectively

Under the microscope, the sublingual gland was mainly composed by acini and striated ducts of the four groups, and the acini cell including the mucous and serous salivary epithelial cells. The cytoplasm of mucous cells was non-staining, with a light red or pink cell membrane, and the cytoplasm of serous cells was stained light purple. Several mucous cells in a circle formed an acinus, surrounded by the serous acinar cells, which resembled a semilunar structure. In the striated ducts, the cell cytoplasm was light red, with plasma membrane infoldings which had red and white microgrooves in the basement membrane. All nuclei were blue or purple. In the SHAM group, the acini were plump and arranged close together without obvious gaps (Fig. 1a). However, in the OVX group, the acini were shriveled and had broader gaps between them (Fig. 1b). Compared to the OVX group, both therapies reversed this sparse arrangement and the presence of spaces (Fig. 1c, d). The percentage of the gap area was higher in the OVX group than in the SHAM group (p < 0.05), whereas it was lower in the OVX + E2 group and OVX + iCR group compared to the OVX group (p < 0.05). When the two treatment groups were compared with the SHAM group, the OVX + E2 group was higher (p < 0.05), but not the OVX + iCR group (p > 0.05). There was no significant difference between the two treatment groups (p > 0.05) (Fig. 2).

### Transmission electron microscopy

Under the electron microscope, the sublingual gland structures of the four groups were observed, including the striated ducts, and mucous and serous salivary epithelial cells.

#### Mucous acinar cells

**Fig. 3:**
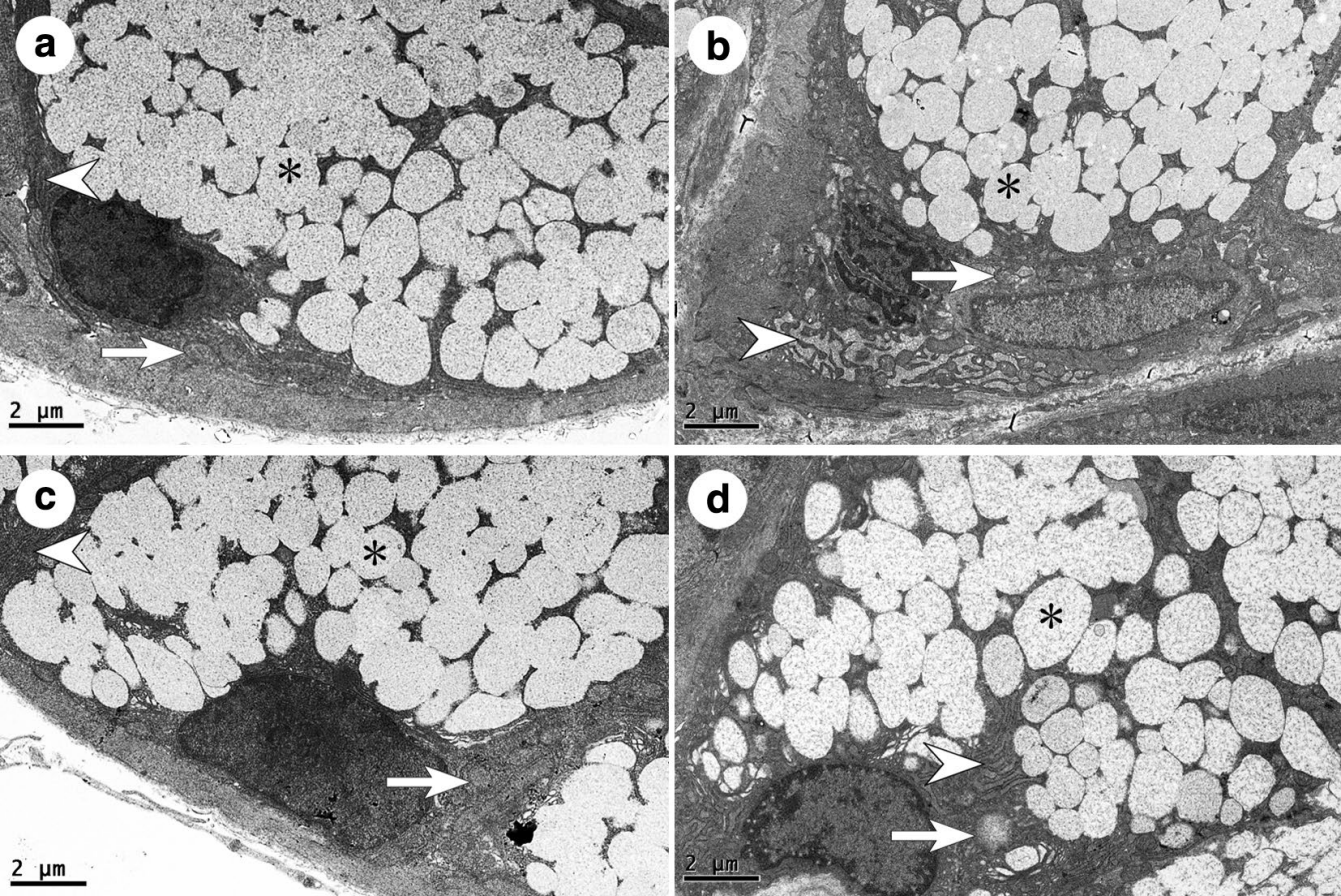
The mucous acinar cells of four groups. **a** SHAM group; **b** OVX group; **c** OVX + E2 group; **d** OVX + iCR group. In picture **a**, the cell nucleus, mitochondria and rough endoplasmic reticulum were arranged in an orderly manner at the basal side of the cell, while secretory granules were located on the luminal side. Nuclear condensation, mitochondrial damage, disordered arrangement of the rough endoplasmic reticulum and decreased numbers of secretory granules was existed in **b**. Almost all the above phenomena were improved appreciably in **c**, **d**. But the mitochondrial damage was still seen in **c**, but not in **d**. *Asterisk* indicates the secretory granules. *White arrows* indicate mitochondria. *White arrow heads* indicate the rough endoplasmic reticulum. *Bars* 2 μm

**Fig. 4:**
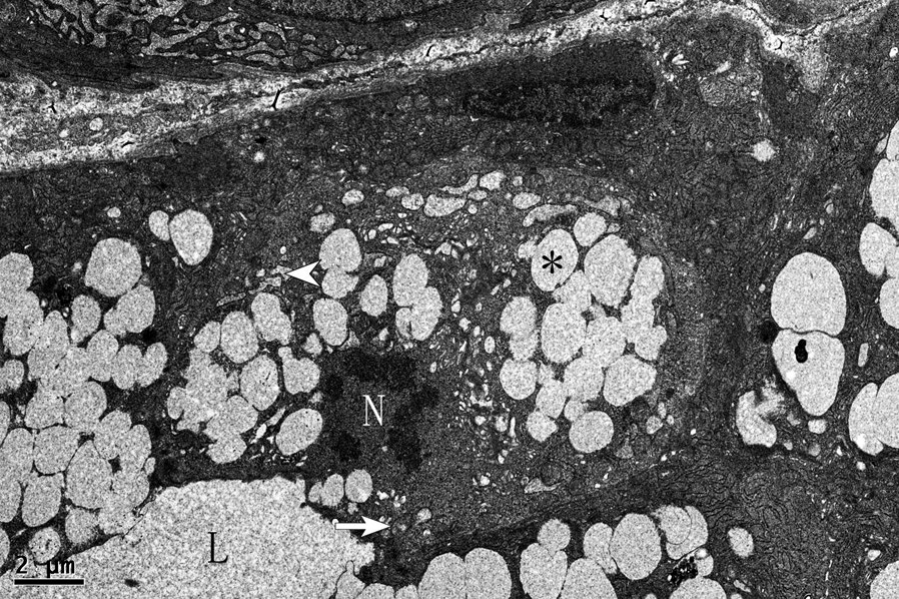
The apoptosis mucous acinar cell of the OVX group. The apoptosis mucous acinar cell was characterized by the disappearance of the nuclear membrane, chromatin pyknosis, decreased secretory granule numbers and changes to cell polarity. *Asterisks* indicates the secretory granules, *N* indicates the nucleus, *L* indicates the lumen. *Bars* 2 μm

The cell nucleus, mitochondria and rough endoplasmic reticulum in the SHAM group were arranged in an orderly manner at the basal lateral membrane of the cell, while secretory granules were located on the luminal side (Fig. 3a). In the OVX group, compared with the SHAM group, there were changes such as nuclear condensation, mitochondrial damage, disordered arrangement of the rough endoplasmic reticulum and decreased numbers of secretory granules (Fig. 3b). Many of the cells were shriveled, with the cell membrane concave to the cytoplasm (data not shown). Also, apoptosis cells in the OVX group increased appreciably, characterized by the disappearance of the nuclear membrane, chromatin pyknosis, decreased secretory granule numbers and changes to cell polarity (Fig. 4). Within the two treatment groups, almost all the above phenomena were improved appreciably. The nuclear condensation was not obvious. The disordered arrangement of the rough endoplasmic reticulum and the decreased secretory granule size were less marked. The only difference between the two groups was in the extent of mitochondrial damage. In the OVX + E2 group, mitochondrial damage was rarely seen, but it was common in the OVX + iCR group (Fig. 3c, d).

#### Serous acinar cells

**Fig. 5:**
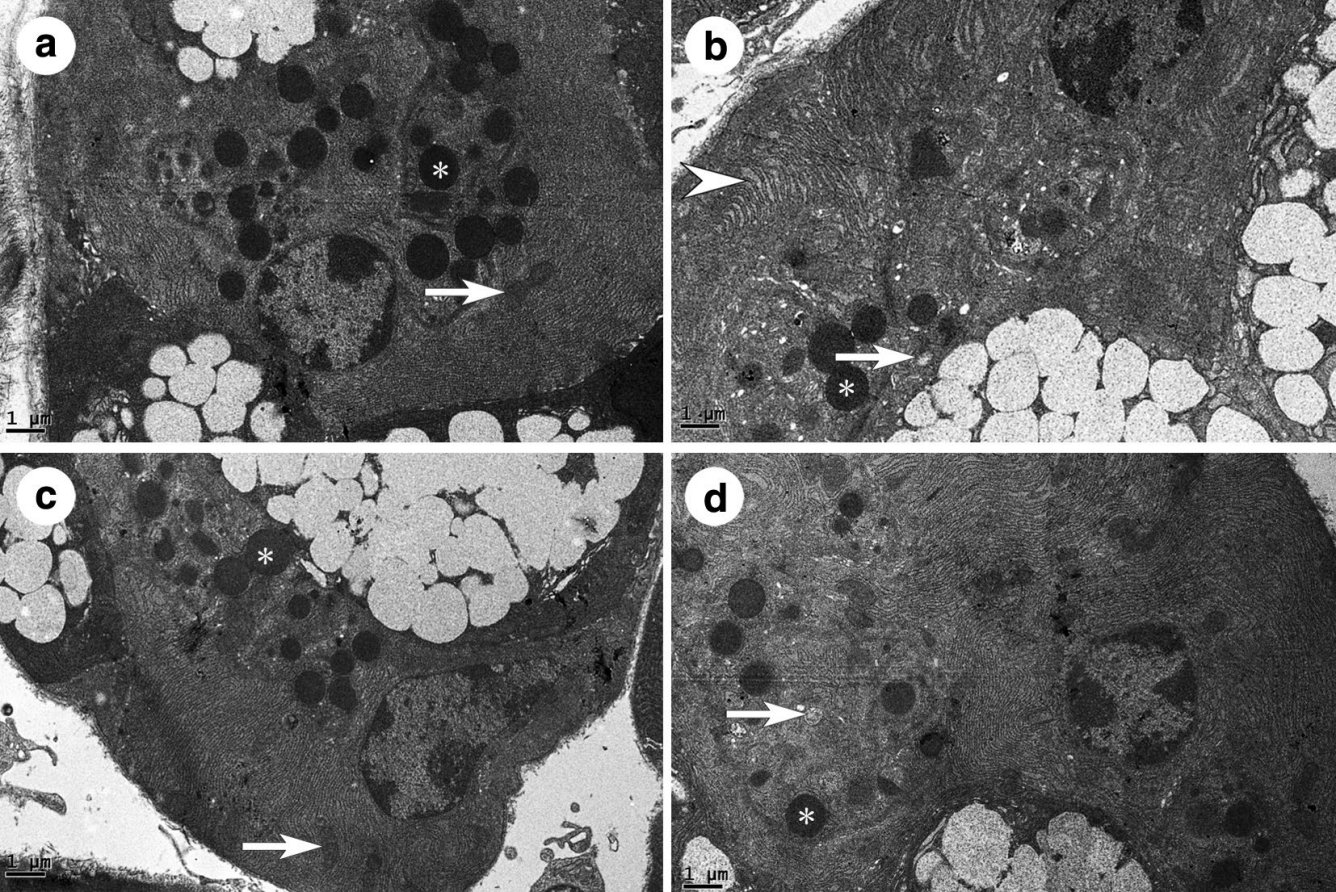
Serous acinar cells of four groups. **a** SHAM group; **b** OVX group; **c** OVX + E2 group; **d** OVX + iCR group. The rough endoplasmic reticulum was arranged like the fingerprint, with the nucleus, mitochondria and black secretory granules embedded in it in **a**. In picture **b**, the rough endoplasmic reticulum arrangement was sparser; the mitochondrial lesions were conspicuous with broken ridges, and the secretory granules had declined in number and volumes. In picture **c**, the rough endoplasmic reticulum was arranged orderly and the mitochondria were healthy. But the number of secretory granules was decreased. In **d**, the rough endoplasmic reticulum seemed normal, but mitochondrial lesions could still be found. The number of secretory granules was declined with less dense coloration. *Asterisk* indicates the secretory granules. *White arrows* indicate the mitochondria. *White arrow heads* indicate the rough endoplasmic reticulum. *Bars* 1 μm

In the SHAM group, the rough endoplasmic reticulum was arranged in a close and orderly manner, like a fingerprint, with the nucleus, mitochondria and black secretory granules embedded in it (Fig. 5a). However, in the OVX group, the rough endoplasmic reticulum arrangement was sparser, with wider gaps. Also, mitochondrial lesions were conspicuous, with broken ridges. The secretory granules had declined in number and volumes (Fig. 5b). Apoptosis could also be commonly observed in the OVX group; the major evidence was nuclear membrane separation, chromatin condensation and cell membrane caveolae (picture not shown). The results of the OVX + E2 group were most similar to those of the SHAM group; the rough endoplasmic reticulum was arranged in an orderly manner and the mitochondria were healthy. But the number of secretory granules was decreased (Fig. 5c). After treatment with black cohosh, the rough endoplasmic reticulum seemed normal, but mitochondrial lesions could still be found. The number of secretory granules was intermediate between the OVX + E2 group and the OVX group, with less dense coloration, but their diameters appeared closer to those of the SHAM group (Fig. 5d).

#### Epithelial cells of the striated ducts

**Fig. 6:**
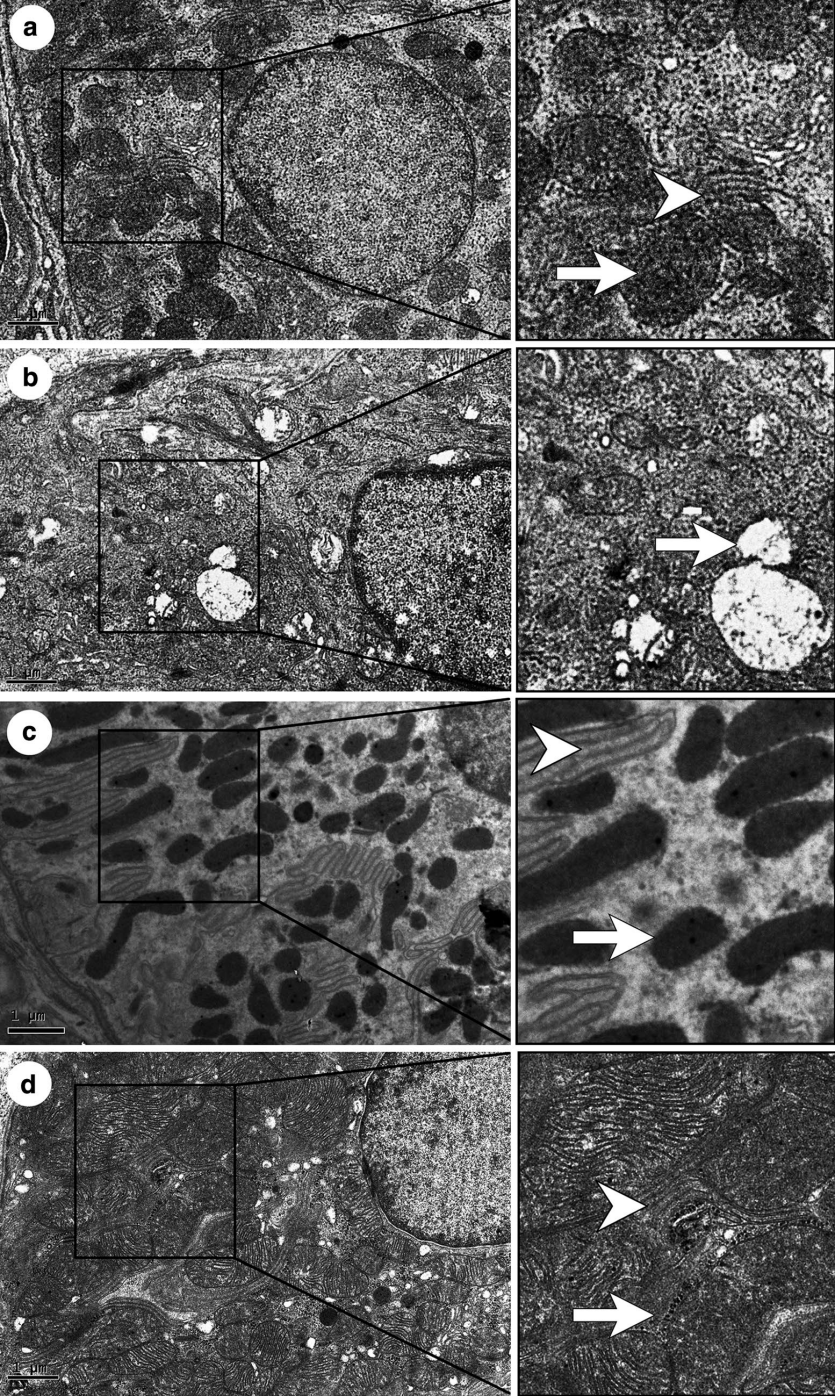
Striated ducts of the sublingual gland. **a** SHAM group; **b** OVX group; **c** OVX + E2 group; **d** OVX + iCR group. In picture **a**, the mitochondria were arranged in the plasma membrane infoldings and integral with its structure. In contrast, the plasma membrane infoldings had been lost, and many mitochondria were damaged, with a double membrane structure and crest lesions in **b**. Mitochondrial damage was rarely found, and plasma membrane infoldings were clearly visible in **c**. The mitochondria were intact in **d**, but they appeared bigger than in the other treatment groups. *White arrows* indicate mitochondria. *White arrow heads* indicate the plasma membrane infoldings. *Bars* 1 μm

In the SHAM group, the mitochondria were arranged in the plasma membrane infoldings and integral with its structure (Fig. 6a). In contrast, the plasma membrane infoldings of the OVX group had been lost, and many mitochondria were damaged, with a double membrane structure and crest lesions (Fig. 6b). Some had also swelled. As with the SHAM group, mitochondrial damage in the OVX + E2 group was rarely found, and plasma membrane infoldings were clearly visible (Fig. 6c). The mitochondria of the OVX + iCR group were intact, but they appeared bigger than in the other treatment groups (Fig. 6d).

### The protein levels of caspase-3

**Fig. 7:**
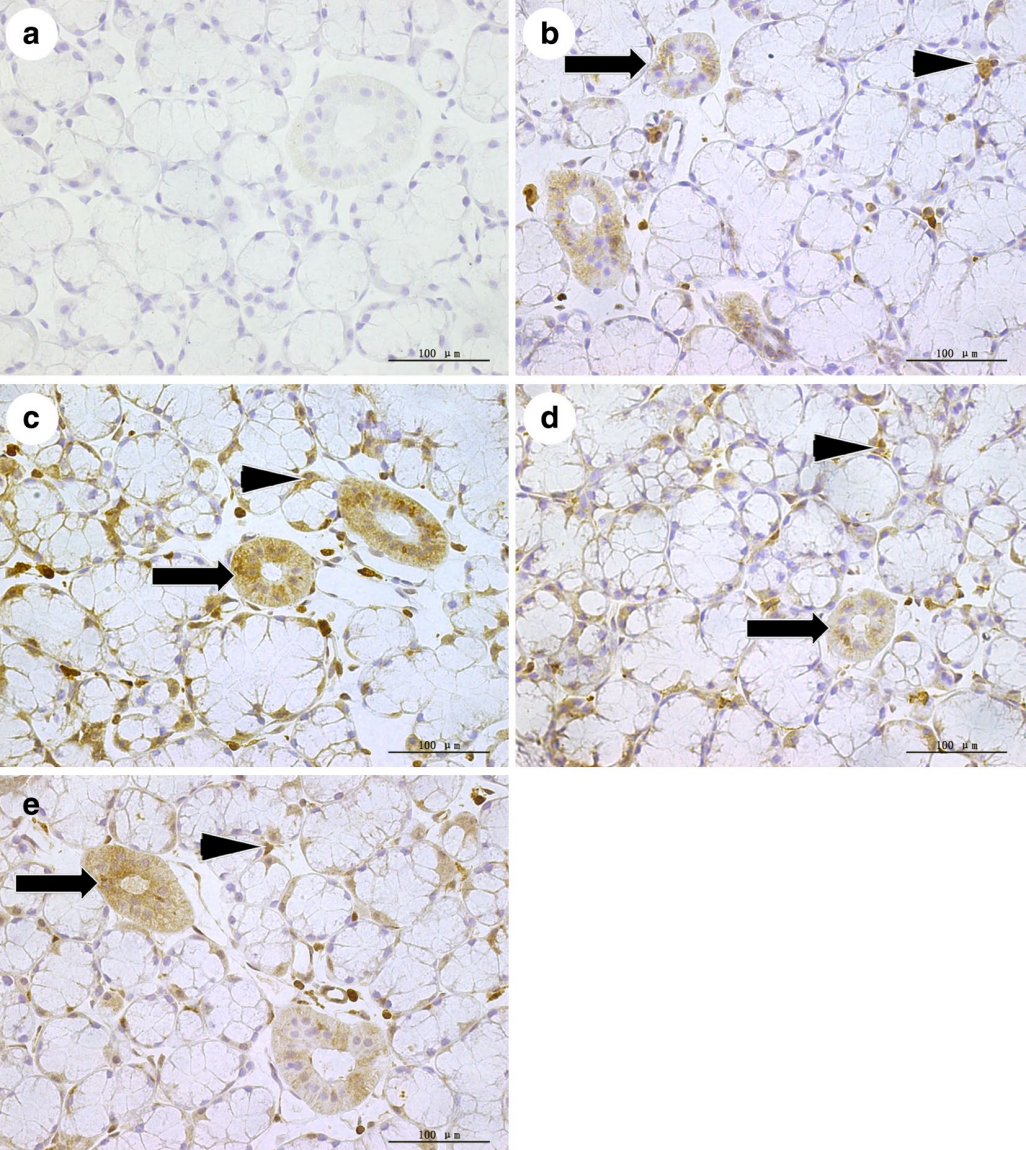
Positive and negative control stainings for caspase-3. **a** Negative control; **b** SHAM group; **c** OVX group; **d** OVX +E2 group; **e** OVX + iCR group. The positive staining was *brown*, could be observed in the cytoplasm of acinar and the striated ducts epithelial cells. *Black arrow heads* indicate the positive cells. *Black arrows* indicate the striated ducts. *Bar* 100 μm

**Fig. 8:**
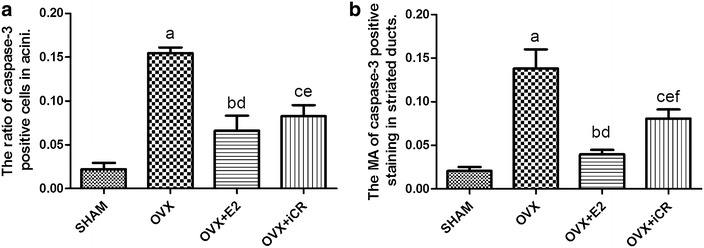
The protein levels of caspase-3 of four groups. **a** The ratio of caspase-3 positive cells in acini of four groups; **b** The MA of caspase-3 positive staining in striated ducts of four groups. Values are given as mean ± SD. ^a,b,c^p < 0.05 for SHAM vs. OVX, OVX + E2 and OVX + iCR respectively. ^d,e^p < 0.05 for OVX vs. OVX + E2 and OVX + iCR, respectively. ^f^p < 0.05OVX + E2 vs. OVX + iCR

Brown color representing positive staining could be observed in the cytoplasm of acinar epithelial cells and epithelial cells of the striated ducts (Fig. 7). The proportion of caspase-3 positive cells was higher in the OVX group than in the SHAM group (p < 0.05), whereas in the OVX + E2 group and in the OVX + iCR group were lower than in the OVX group (p < 0.05), but higher than in SHAM group (p < 0.05). There was no significant difference between the two drug treatment groups (Fig. 8a). In the striated ducts, the statistics for MA showed a significant increase in the OVX group and the OVX + iCR group compared with the SHAM group (p < 0.05). The MA of the OVX + E2 group decreased in contrast to the OVX group and the OVX + iCR group (p < 0.05). The MA of caspase-3 was also reduced by iCR therapy lower than in OVX group (p < 0.05), but the effect appeared to be less than that of estrogen (p < 0.05) (Fig. 8b).

## Discussion

According to the two sections of the hypothesis proposed by Thaysen [17], salivary gland acinar cells firstly secrete isotonic liquid, primary saliva, which is rich in Na^+^ and Cl^−^ ions. Then, primary saliva reabsorbs Na^+^ and Cl^−^ across the duct system, secreting HCO3^−^ and K^+^, but does not cause the removal of water, ultimately forming hypoosmotic saliva. This hypothesis indicates that the formation of saliva is closely related to the normal functioning of the acinar and ductal systems. The present study was performed on ovariectomized rats in order to simulate the state of the salivary glands in postmenopausal women, and found that removing the ovaries led to the lesions of both acinar and ductal systems, including atrophy of acinar cells, apoptosis, and lesions of cellular organelles and up-regulation of the level of caspase-3. However, estrogen could alleviate these morphological changes and down-regulate expression of the apoptosis marker caspase-3. Black cohosh treatment could have a similar effect to estrogen, but the degree of effect and the specific action were not the same. In the mitochondria of the striated ducts, there was no obvious crest fracture, but volume increased obviously in the OVX + iCR group. Mitochondria with increased volume may be a manifestation of drug poisoning, eventually affecting their functioning. This inhibiting effect of caspase-3 expression was also inferior to that of estrogen on the striated ducts. It is of interest that there were no significant differences in HE staining between the two treatment groups, which may prompt the notion that black cohosh plays a further role, such as resistance to oxidative stress, or a protective effect on a dominant nerve, so that the HE staining is similar in both groups.

Generally, cell atrophy has several impact factors caused or aggravated it, including decreased functional activity of cells, and a lack of blood supply and nutrients to nerves. These impact factors could be caused by a decrease in endocrine stimulation. One previous study, similarly to ours, found that the use of estrogen or estrogen and soy isoflavone combination therapy had a therapeutic effect on ductal cells and acinar atrophy in ovariectomized rats [18]. Recently it has been demonstrated that black cohosh does not have estrogen-like effects and does not bind to estrogen receptors [19]. Further, it could not increase estrogen-dependent gene expression, and did not stimulate the growth of estrogen-dependent tumors. However its therapeutic effect on osteoporosis, hot flashes and other postmenopausal symptoms were similar to those of estrogen. So, the anti-atrophy effect of black cohosh may be due to its estrogen-like effects. The exact mechanism underlying these effects is worthy of further study.

Under the transmission electron microscope, the main changes after ovariectomy can be summarized as follows: (1) change to the dilation of the rough endoplasmic reticulum; (2) damage to the integrity of mitochondria; (3) apoptosis; (4) disappearance of plasma membrane infoldings within the striated ducts.

The rough endoplasmic reticulum is the site of synthesis of secreted proteins and structural proteins destined for the plasma membranes; most secreted proteins and transmembrane proteins are folded here, acquiring their natural conformations. It was well known that the relationship of estrogen and estrogen receptor (ER) is mutual. Estrogen depends on the presence of ERs to have an effective action, and the decline in estrogen following ovariectomy also can down-regulate the synthesis of ER. Using the colloidal gold technique to label pituitary cells, the expression of estrogen receptor alpha (ERα) and estrogen receptor beta (ERβ) has been detected in the lumen of the rough endoplasmic reticulum, provided the condition for the role of estrogen [20]. There is evidence to suggest that endoplasmic reticulum stress can induce the expression of caspase-3, triggering apoptosis called endoplasmic reticulum stress–induced apoptosis pathway, whereas estrogen concentrations can counteract Akt deactivation caused by endoplasmic reticulum stress through binding to ERs, reduced the expression of endoplasmic reticulum stress markers, thereby inhibiting endoplasmic reticulum stress-induced apoptosis pathway [21–23]. Nothing in the literature can account for the mechanism, by which black cohosh inhibits rough endoplasmic reticulum expansion. It may be due directly to its anti-apoptotic effect.

The most important role of mitochondria is to convert chemical energy to ATP (Adenosine Triphosphate), a function which is closely related to many cellular activities. At the same time, mitochondria play a key role in the process of apoptosis. Research on myocardial cells found that an active pool of P38 beta existed in the mitochondria, which can activate manganese superoxide dismutase (MnSOD) (a critical part of the mitochondrial antioxidant system) and this process is estrogen-dependent. Estrogen was found to protect myocardial cells, possibly by reducing mitochondrial oxidative stress [24]. According to the electron microscope observations of mitochondria in the current work, we hypothesize that estrogen may play a similar role in the sublingual gland. It has been found that deoxyactein extract from black cohosh inhibits the dissipation of mitochondrial membrane potential and cardiolipin oxidation, and decreases the release of reactive oxygen species and 3-nitrotyrosine. The study showed that deoxyactein might help to protect mitochondria from rupture due to oxidative stress. So, black cohosh played a role in protecting mitochondria, possibly through substances such as deoxyactein [25].

The main function of plasma membrane infoldings is to increase the surface area of basal cells, which aids with rapid water and electrolyte transport, and so it is of importance to the process of saliva formation. It has not been previously reported that ovariectomy leads to the disappearance of plasma membrane infoldings on salivary gland ductal system. The two drugs treatments used here could ameliorate this situation. It has been demonstrated that knock-out of ERβ, which is the dominate estrogen receptor in the salivary gland ductal system; can cause reduced expression of the N-Myc proto-oncogene protein downstream of the protein coding gene, NDRG2. The protein coded by NDRG2 mainly exists in the cytoplasm of salivary gland ductal cells, combining and stabilizing the Na, K-ATPase beta 1 protein, which plays a key role in ion transport. Na, K-ATPase beta 1 protein is abundant in plasma membrane infoldings. Hence, we suggest that protection of the infoldings by estrogen might relate to the NDRG2 gene [26]. How black cohosh produces an effect is unclear. Much research has classified black cohosh as a selective estrogen receptor agonist, meaning it would act as an estrogen in many respects; this needs further exploration.

The apoptotic cascade reaction which activates caspase-3 was found to be able to have an impact on the structural transformation of apoptotic cells, so DNA damage and cell atrophy are both caspase-3 activation dependent. Therefore, caspase-3 has been considered to be a specific immunohistochemical marker of apoptosis [27, 28]. In the mitochondria-mediated apoptosis pathway, the mitochondrial membrane was damaged and resultant cytochrome-c was released, then the apoptosis cascades were triggered, and caspase-3 was considered to be the terminal factor, marking the end stage of apoptosis [29]. Studies have shown that ovariectomy could lead to cell apoptosis in some tissues, including salivary gland [14]. But a research of small salivary gland cells in vitro found that lack of estrogen was not the cause of salivary gland cell apoptosis [30]. Although the activity of caspase-3 was not assessed in the study, the higher protein levels of caspase-3 herald that the apoptosis is occurring. In addition, we observed lots of apoptosis cells of OVX group under the electron microscope. Mutual authentication, using the caspase-3 level and apoptosis cells under the electron microscope, further confirms the existence of apoptosis. Results from Fig. 7 indicate a lower effect of E2 on caspase-3 levels when compared to OVX rats, but not with control group, which clearly support the idea that estrogen can inhibit apoptosis. In recent years, the results of several studies have suggested that the anti-apoptotic effects of estrogen occur mainly through a non-genomic mechanism. This non-genomic mechanism of action of estrogen is usually connected with the activation of the cytoplasmic protein kinase system, which includes the mitogen-activated protein kinase (MAPK) system [EPK1/2-dependent signaling cascade, c-Jun N-terminal kinases (JNK) pathway, PI3K activation (PI3K/Akt; protein kinase B, PKB) pathway] among others. Further studies are required to find out which pathway plays a role in the salivary glands. Although the specific mechanism remains unclear, the anti-apoptotic effect of black cohosh might generally be related to its anti-aging, anti-oxidation and estrogen-like effect.

As an initial exploration into the effects of black cohosh on the sublingual gland, the present study has limitations. In order to enhance the curative effect and provide clinical medication guidance, further studies are required. Firstly, the exact mechanisms by which estrogen and black cohosh influence the rough endoplasmic reticulum, mitochondria and plasma membrane infoldings require exploration. Secondly, further efforts are required to study and compare the similarities and differences between the effects of the two medicines on salivary gland secretions and their ionic and protein contents. Thirdly,for the alteration in the mitochondrial size and lower inhibition of caspase-3 was presented in the OVX + iCR animals, it is worth to explore whether there were some long-term damages appear in the sublingual gland in these animals. Finally, due to the complex chemical composition of black cohosh, it will be helpful to distinguish exactly which components have positive effects on the acinar cells and ducts of sublingual glands.

## Conclusion

The current study identified ovariectomy caused injuries in the subcellular structure and cell apoptosis of the sublingual glands. Estrogen and *C. racemosa* decreased the lesions, but the specific effects and mechanisms were not identical. Also, it seemed like the efficacy of *C. racemosa* inferior to estrogen, and probably did not act through estrogen receptors. This study promotes the experimental study of black cohosh and the medication of women in the perimenopausal period.

## Additional files

**Additional file 1.** The percentage of the gap area in sublingual gland of four groups.

**Additional file 2.** The ratio of caspase-3 positive cells in sublingual gland of four groups.

**Additional file 3.** The MA of caspase-3 in straited ducts in sublingual gland of four groups.

## References

[1] Evans MP, Fleming KC, Evans JM (1995). Hormone replacement therapy: management of common problems. Mayo Clin Proc.

[2] Eliasson L, Carlén A, Laine M (2003). Minor gland and whole saliva in postmenopausal women using a low potency oestrogen (oestriol). Arch Oral Biol.

[3] Kupferer EM, Dormire SL, Becker H (2009). Complementary and alternative medicine use for vasomotor symptoms among women who have discontinued hormone therapy. J Obstet Gynecol Neonatal Nurs.

[4] Carpenter JS, Neal JG (2005). Other complementary and alternative medicine modalities: acupuncture, magnets, reflexology, and homeopathy. Am J Med.

[5] Carroll DG (2006). Nonhormonal therapies for hot flashes in menopause. Am Fam Physician.

[6] Nedrow A, Miller J, Walker M (2006). Complementary and alternative therapies for the management of menopause-related symptoms: a systematic evidence review. Arch Intern Med.

[7] Rees M (2009). Alternative treatments for the menopause. Best Pract Res Clin Obstet Gynaecol.

[8] Tiran D (2006). Integrated healthcare: herbal remedies for menopausal symptoms. Br J Nurs.

[9] Bai W, Henneicke-von Zepelin HH, Wang S (2007). Efficacy and tolerability of a medicinal product containing an isopropanolic black cohosh extract in Chinese women with menopausal symptoms: a randomized, double blind, parallel-controlled study versus tibolone. Maturitas.

[10] Cui G, Leng H, Wang K (2013). Effects of remifemin treatment on bone integrity and remodeling in rats with ovariectomy-induced osteoporosis. PLoS ONE.

[11] Burdette JE, Chen S, Lu ZZ (2002). Black cohosh (*Cimicifuga racemosa* L.) protects against menadione-induced DNA damage through scavenging of reactive oxygen species: bioassay-directed isolation and characterization of active principles. J Agric Food Chem.

[12] Moon L, Ha YM, Jang HJ (2011). Isoimperatorin, cimiside E and 23-O-acetylshengmanol-3-xyloside from *Cimicifugae rhizome* inhibit TNF-α-induced VCAM-1 expression in human endothelial cells: involvement of PPAR-γ upregulation and PI3 K, ERK1/2, and PKC signal pathways. J Ethnopharmacol.

[13] Ma X, Zhang H, Wang K (2011). Effects of an isopropanolic-aqueous black cohosh extract on central body temperature of ovariectomized rats. J Ethnopharmacol.

[14] Da Y, Niu K, Wang K (2015). A comparison of the effects of estrogen and *Cimicifuga racemosa* on the lacrimal gland and submandibular gland in ovariectomized rats. PLoS ONE.

[15] Rachon D, Vortherms T, Seidlová-Wuttke D (2008). Effects of black cohosh extract on body weight gain, intra-abdominal fat accumulation, plasma lipids and glucose tolerance in ovariectomized Sprague–Dawley rats. Maturitas.

[16] Wang W, Bai W, Cui G (2015). Effects of estradiol valerate and remifemin on norepinephrine signaling in the brain of ovariectomized rats. Neuroendocrinology.

[17] Thaysen JH, Thorn NA, Schwartz IL (1954). Excretion of sodium, potassium, chloride and carbon dioxide in human parotid saliva. Am J Physiol.

[18] Carvalho VDC, Silveira VÁS, do Prado RF (2011). Effect of estrogen therapy, soy isoflavones, and the combination therapy on the submandibular gland of ovariectomized rats. Pathol Res Pract.

[19] Mahady GB (2003). Is black cohosh estrogenic?. Nutr Rev.

[20] González M, Reyes R, Damas C (2008). Oestrogen receptor alpha and beta in female rat pituitary cells: an immunochemical study. Gen Comp Endocrinol.

[21] Fu Z, Zou F, Deng H (2014). Estrogen protects SGC7901 cells from endoplasmic reticulum stress-induced apoptosis by the Akt pathway. Oncol Lett.

[22] Kozlov AV, Duvigneau JC, Hyatt TC (2010). Effect of estrogen on mitochondrial function and intracellular stress markers in rat liver and kidney following trauma-hemorrhagic shock and prolonged hypotension. Mol Med.

[23] Salmans ML, Zhao F, Andersen B (2013). The estrogen-regulated anterior gradient 2 (AGR2) protein in breast cancer: a potential drug target and biomarker. Breast Cancer Res.

[24] Liu H, Yanamandala M, Lee TC (2014). Mitochondrial p38β and manganese superoxide dismutase interaction mediated by estrogen in cardiomyocytes. PLoS ONE.

[25] Choi EM (2013). Deoxyactein Isolated from *Cimicifuga racemosa* protects osteoblastic MC3T3-E1 cells against antimycin A-induced cytotoxicity. J Appl Toxicol.

[26] Li Y, Liu C, Hou W (2013). Retrograde ductal administration of the adenovirus-mediated gene leads to improved sialaden hypofunction in estrogen-deficient rats. Mol Ther..

[27] Hughes J, Gobe G (2007). Identification and quantification of apoptosis in the kidney using morphology, biochemical and molecular markers. Nephrology.

[28] Kousteni S, Han L, Chen JR (2003). Kinase-mediated regulation of common transcription factors accounts for the bone-protective effects of sex steroids. J Clin Investig.

[29] Wang Q, Zhang L, Yuan X (2016). The relationship between the Bcl-2/Bax proteins and the mitochondria-mediated apoptosis pathway in the differentiation of adipose-derived stromal cells into neurons. PLoS ONE.

[30] Tsinti M, Kassi E, Korkolopoulou P (2009). Functional estrogen receptors alpha and beta are expressed in normal human salivary gland epithelium and apparently mediate immunomodulatory effects. Eur J Oral Sci.

